# KDM4 inhibitor SD49-7 attenuates leukemia stem cell *via* KDM4A/MDM2/p21^CIP1^ axis

**DOI:** 10.7150/thno.71460

**Published:** 2022-06-21

**Authors:** Yinghui Li, Chaoqun Wang, Huier Gao, Jiali Gu, Yiran Zhang, Yingyi Zhang, Min Xie, Xuelian Cheng, Ming Yang, Wenshan Zhang, Yafang Li, Mei He, Hui Xu, Hexiao Zhang, Qing Ji, Tianhua Ma, Sheng Ding, Yu Zhao, Yingdai Gao

**Affiliations:** 1 State Key Laboratory of Experimental Hematology, National Clinical Research Center for Blood Diseases, Haihe Laboratory of Cell Ecosystem, PUMC Department of Stem Cell and Regenerative Medicine, CAMS Key Laboratory of Gene Therapy for Blood Diseases, Institute of Hematology & Blood Diseases Hospital, Chinese Academy of Medical Sciences & Peking Union Medical College, Tianjin 300020, China.; 2 Department of Pharmacy, Tianjin First Central Hospital, School of Medicine, Nankai University, Tianjin 300192, China.; 3 Department of Biochemistry and Molecular Biology, Mayo Clinic, Rochester, MN 55901, USA.; 4 Gladstone Institute of Neurological Disease, San Francisco, CA 94158, USA.; 5 School of Pharmaceutical Sciences, Tsinghua University, Beijing 100084, China.; 6 Tsinghua-Peking Joint Center for Life Sciences, Tsinghua University, Beijing 100084, China.; 7 Tianjin Key Laboratory of Radiation Medicine and Molecular Nuclear Medicine, Institute of Radiation Medicine, Chinese Academy of Medical Sciences and Peking Union Medical College, Tianjin 300192, China.

**Keywords:** Leukemia stem cells, Lysine-specific demethylase 4, Small molecular compounds

## Abstract

**Rationale:** Traditional treatments for leukemia fail to address stem cell drug resistance characterized by epigenetic mediators such as histone lysine-specific demethylase 4 (KDM4). The KDM4 family, which acts as epigenetic regulators inducing histone demethylation during the development and progression of leukemia, lacks specific molecular inhibitors.

**Methods:** The KDM4 inhibitor, SD49-7, was synthesized and purified based on acyl hydrazone Schiff base. The interaction between SD49-7 and KDM4s was monitored *in vitro* by surface plasma resonance (SPR). *In vitro* and *in vivo* biological function experiments were performed to analyze apoptosis, colony-formation, proliferation, differentiation, and cell cycle in cell sub-lines and mice. Molecular mechanisms were demonstrated by RNA-seq, ChIP-seq, RT-qPCR and Western blotting.

**Results:** We found significantly high *KDM4A* expression levels in several human leukemia subtypes. The knockdown of KDM4s inhibited leukemogenesis in the MLL-AF9 leukemia mouse model but did not affect the survival of normal human hematopoietic cells. We identified SD49-7 as a selective KDM4 inhibitor that impaired the progression of leukemia stem cells (LSCs) *in vitro*. SD49-7 suppressed leukemia development in the mouse model and patient-derived xenograft model of leukemia. Depletion of KDM4s activated the apoptosis signaling pathway by suppressing *MDM2* expression *via* modulating H3K9me3 levels on the *MDM2* promoter region.

**Conclusion:** Our study demonstrates a unique KDM4 inhibitor for LSCs to overcome the resistance to traditional treatment and offers KDM4 inhibition as a promising strategy for resistant leukemia therapy.

## Introduction

Acute myeloid leukemia (AML) is a malignant disorder characterized by abnormal growth and differentiation of hematopoietic stem/progenitor cells (HSPCs) 1. It is the most common acute leukemia type, accounting for approximately 80% of acute leukemias in adults 2, with < 50% long-term overall survival rates for younger patients and < 1 year for older patients 3. Leukemia stem cells (LSCs) are a low-frequency subpopulation of leukemia cells in AML that possess stem cell properties, including self-renewal capacity and drug resistance, which purportedly facilitate the development and relapse of AML 4. Thus, elucidating the regulatory mechanism of LSCs is believed to be a crucial barrier to overcoming leukemia. Generally, AML is caused by chromosome aberrations, such as translocations, inversions, and deletions. A burgeoning body of evidence has shown that epigenetic alterations are key players in the development of most AMLs and points to the exploitation of new molecular targets for more efficacious therapies 5. However, the mechanisms of epigenetic regulation in LSCs remain unclear.

Methylation of histone lysine residues, which can selectively activate or silence gene expression, is important for epigenetic information and is closely related to AML development 6. There are two histone demethylase protein families, the lysine demethylases (LSDs) and the JMJC domain-containing demethylases with different functional mechanisms, and the dysfunction of either family can contribute to AML development 7. It has been reported that LSD1 is necessary for maintaining the stemness of LSCs induced by the MLL-AF9 rearrangement in AML 8. The JMJC domain-containing demethylase family has seven subfamilies related to the initiation and maintenance of various malignant tumors, including AML 9. The JMJD2/KDM4 protein family consists of four members:* KDM4A*, *KDM4B*, *KDM4C*, and *KDM4D*. These proteins are highly homologous except for *KDM4D*, which lacks the C-terminal part of the protein 10. It has been reported that either direct depletion of* Kdm4c* or using a KDM4C inhibitor, SD70, can successfully suppress the transcription and transformation ability of MLL fusions in leukemia cells 11, 12. Wang et al. reported that leukemogenic chromatin alterations promote AML leukemia stem cells *via* a KDM4C-ALKBH5-AXL signaling axis 13, suggesting that KDM4C is a key regulator in leukemogenesis. However, Agger et al. reported that inhibition of KDM4C only was not sufficient for suppressing leukemia progression, but knockout of *Kdm4a, b,* and* c* could block leukemia progression 14. These results indicated that KDM4 is a potential therapeutic target and developing new drugs targeting KDM4 is a promising option for AML treatment. However, the role of different KDM4 members in leukemia progression needs to be further verified.

Traditional clinical chemotherapy drugs that target cell proliferation have little potency in eliminating LSCs because of their quiescent status 15. Similar characteristics of LSCs and hematopoietic stem cells (HSCs) make it challenging to develop drugs to selectively attenuate LSCs with little impact on HSCs. Small molecules that can selectively target LSCs are rare and still in early stages of development, and several natural or constructed compounds have been reported to impair LSCs 16-20. Histone demethylases are known to play a role in regulating LSCs and targeting KDM4C by SD70 could inhibit leukemogenesis in a spontaneous leukemia mouse model 11. However, effective targeted therapy strategies still require a mechanism-specific understanding to identify and validate new agents targeted to LSCs.

In this study, we found that *KDM4A* expression levels were significantly higher in several human leukemia subtypes, and silencing of *Kdm4a* could sufficiently inhibit the leukemogenic ability in an *in vivo* MLL-AF9 leukemia model 21. Compared with the *KDM4C* knockdown, the survival of human CD34^+^ umbilical cord blood cells was rarely affected by knocking down *KDM4A*. Furthermore, we acquired a series of known and/or potential KDM4 inhibitors for screening, and one of these candidates, SD49-7, impaired LSCs by targeting KDM4A but did not affect normal HSCs both in mice and patient-derived tumor xenograft (PDX) AML models. Mechanistically, we found that depletion of KDM4A by either shRNA or SD49-7 activated the apoptosis signaling pathway by inhibiting *MDM2* expression *via* modulating H3K9me3 levels on the *MDM2* promoter region. Our study elucidated the regulatory role of KDM4 in LSCs, implicating the KDM4A/MDM2 pathway in LSC regulation. We also evaluated a novel KDM4 inhibitor that could selectively eradicate LSCs and serve as a promising candidate for leukemia therapy. Our work provided new insights into the mechanism of KDM4 in leukemia regulation and novel ideas for epigenetic drug design to target LSCs.

## Results

### KDM4s are key regulators in leukemia progression

**Figure 1:**
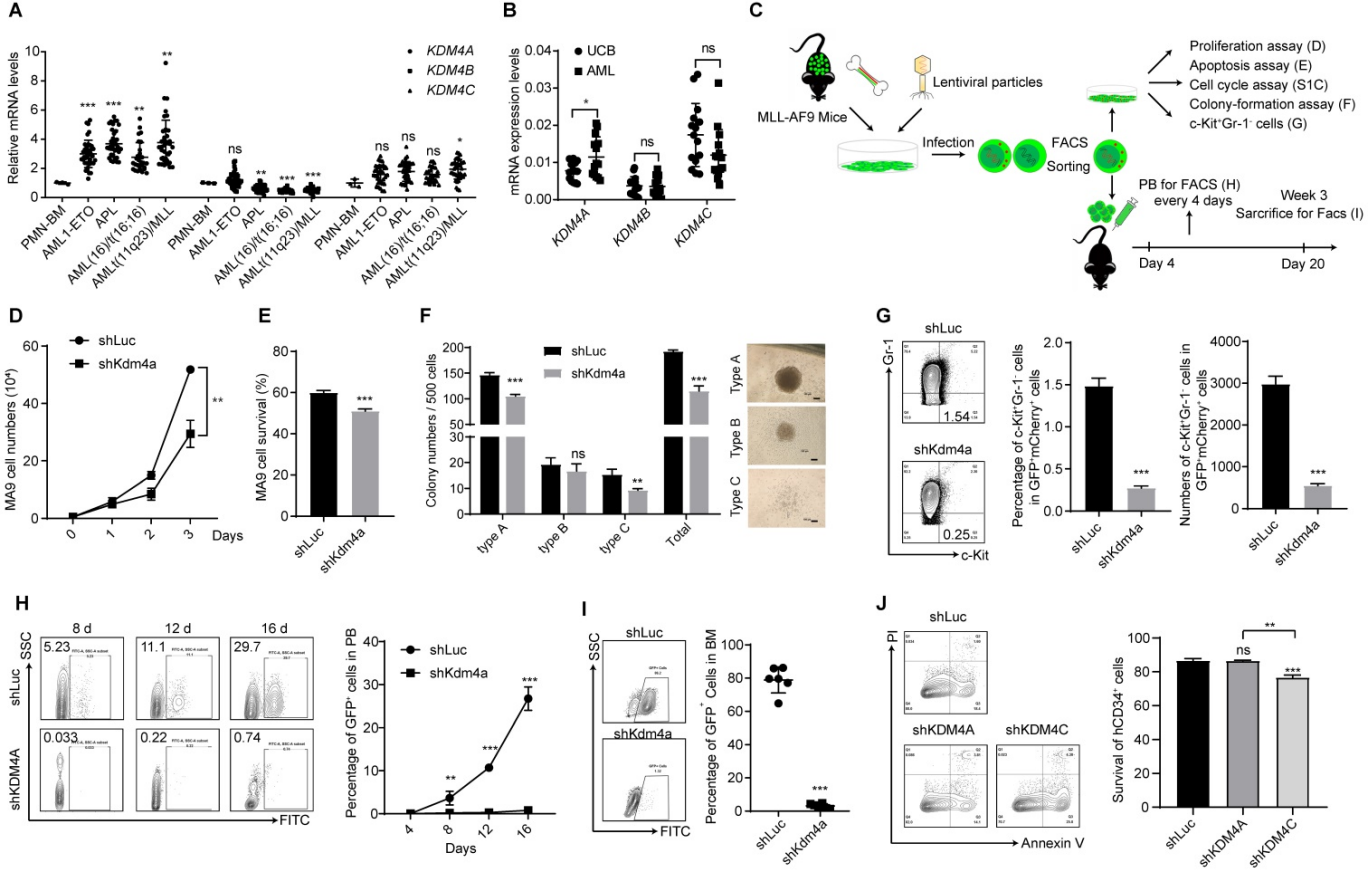
** KDM4 is a key regulator in leukemia progression. (A)** Relative mRNA expression levels of KDM4A, KDM4B, and KDM4C in PMN-BM, AML1-ETO, APL, AML (16)/t(16:16), and AML t(11q23)/MLL. Data were from HemaExplorer (n = 3 in PMN-BM, 39 in AML1-ETO, 37 in APL, 28 in AML (16)/t(16:16), and 38 in AML t(11q23)/MLL, respectively). Data were normalized to PMN-BM. **(B)** mRNA expression levels of KDM4A, KDM4B, and KDM4C in umbilical cord blood and AML patients' bone marrow mononuclear cells (n = 15). **(C)** Workflow of the MLL-AF9 mode- related experimental design. **(D)** MLL-AF9 leukemia cell proliferation in shLuc and shKDM4A groups by cell counting (n = 3). **(E)** Apoptosis of MLL-AF9 leukemia cells in shLuc and shKDM4A groups (n = 3). **(F)** Colony numbers (left) and representative microscopy images of colony formation (right) in shLuc and shKDM4A groups (n = 3). 500 MLL-AF9 leukemia cells per well were seeded into a 24-well plate. Scale bar = 100 µm. **(G)** Percentage (middle panel) and absolute (right panel) numbers of c-Kit^+^Gr-1^-^ cell population of shLuc and shKDM4A MLL-AF9 leukemia cells (n = 3). Representative flow cytometric results are shown in the left panel. **(H)** Percentage of GFP^+^ cells in peripheral blood (PB) on days 4, 8, 12, and 16 was detected by flow cytometry in the *ex vivo* translation model (right panel, n = 3). Representative flow cytometric results are shown in the left panel. **(I)** Percentage of GFP^+^ cells in bone marrow (BM) was detected by flow cytometry in the *ex vivo* translation model (right panel, n = 6). Representative flow cytometric results are shown in the left panel. **(J)** Apoptosis of CD34^+^ UCB cells in shLuc, shKDM4A, and shKDM4C groups (left panel, n = 3). Representative flow cytometric results are shown in the left panel. All experiments were repeated three times. ns. no significance, *p < 0.05, **p < 0.01, ***p < 0.001, by unpaired Student's* t*-test, error bars denote mean ± SD.

KDM4C has been reported to act as a regulator involved in murine AML 13, 22. However, the role of other KDM4 members in AML remains unclear. We investigated the expression of different KDM4 members in various subtypes of AML patients using the gene expression profiling datasets (HemaExplorer, http://servers.binf.ku.dk/hemaexplorer/). Our analysis indicated that KDM4A was expressed at significantly higher levels in four AML subtypes than normal mononuclear cell controls, while KDM4C was expressed at only slightly higher levels in AML with MLL-amplification than in controls. The expression of KDM4B was higher in normal mononuclear cells than in different AML subtypes (Figure 1A). Our data also showed that KDM4A expression levels were higher in cells from AML patients, and the expression levels of KDM4B and KDM4C were comparable between AML patients and normal hematopoietic mononuclear cells from cord blood (Figure 1B).

Upregulation of KDM4A in AML cells prompted us to investigate its function in leukemogenesis using an MLL-AF9 leukemia mouse model (Figure 1C). Bone marrow cells from MLL-AF9 AML mice were harvested, and a short hairpin RNA (shRNA)-mediated knockdown was performed to abrogate KDM4A expression. We used three independent shRNAs to knock down KDM4A expression and selected the most efficient one to perform subsequent experiments (Figure S1A-B). Compared with the control group, silencing of KDM4A significantly inhibited cell proliferation (Figure 1D), induced cell apoptosis (Figure 1E), and slightly accelerated cells into the S phase (Figure S1C). Moreover, the colony formation numbers of leukemia cells, especially the most malignant colony type A 23, were significantly reduced by KDM4A knockdown (Figure 1F).

Next, we examined the variations in the mouse LSCs enriched in the c-Kit^+^ Gr-1^-^ cell population 24. The results showed that the percentage and the absolute number of the c-Kit^+^Gr-1^-^ population were markedly inhibited by KDM4A knockdown (KD) (Figure 1G). Furthermore, transplantation of KDM4A-silenced leukemia cells significantly lowered the percentage of leukemia cells in the peripheral blood of recipient mice (Figure 1H). Also, the GFP^+^ leukemia cells could hardly be detected in the bone marrow (BM) of the recipients of KDM4A KD cells (Figure 1I). These data indicated that, like KDM4C, KDM4A was also required for AML development.

To further investigate KDM4A function in human leukemia cells, we used the human myeloid leukemia cell line THP-1 and performed subsequent experiments using the most efficient of three different shRNAs (Figure S1D-E). Our data showed that KDM4A knockdown significantly induced cell apoptosis (Figure S1F), arrested the cell cycle in G_0_/G_1_ phase (Figure S1G), and up-regulated the expression of the cell differentiation marker CD11b on the cell membrane (Figure S1H). Colony formation assays also showed that knocking down KDM4A reduced the colony numbers of THP-1 cells (Figure S1I), indicating that KDM4A was required for the survival and development of human AML cells.

KDM4C has been reported to act as a regulator in AML 13, and inhibition of *Kdm4c* could inhibit leukemogenesis in a murine leukemia model 11. However, the effect of depleting KDM4 members on normal hematopoietic cells remains unknown. We explored whether KDM4A or KDM4C was required for normal human hematopoietic cells by knocking down KDM4A and KDM4C using shRNAs in human cord blood (UCB)-derived CD34^+^ cells (Figure S1J). The results showed that knocking down KDM4A failed to affect apoptosis of CD34^+^ cells, while KDM4C knockdown could significantly induce apoptosis (Figure 1J). Moreover, knockdown of both KDM4A and KDM4C decreased colony formation ability of UCB-derived CD34^+^ cells (Figure S1K-L). These results indicated that KDM4A might serve as an AML treatment target similar to KDM4C and targeting KDM4s has the potential to significantly inhibit leukemia cells without killing normal human hematopoietic cells.

### SD49-7 is a small molecule inhibitor of KDM4A and KDM4C

**Figure 2:**
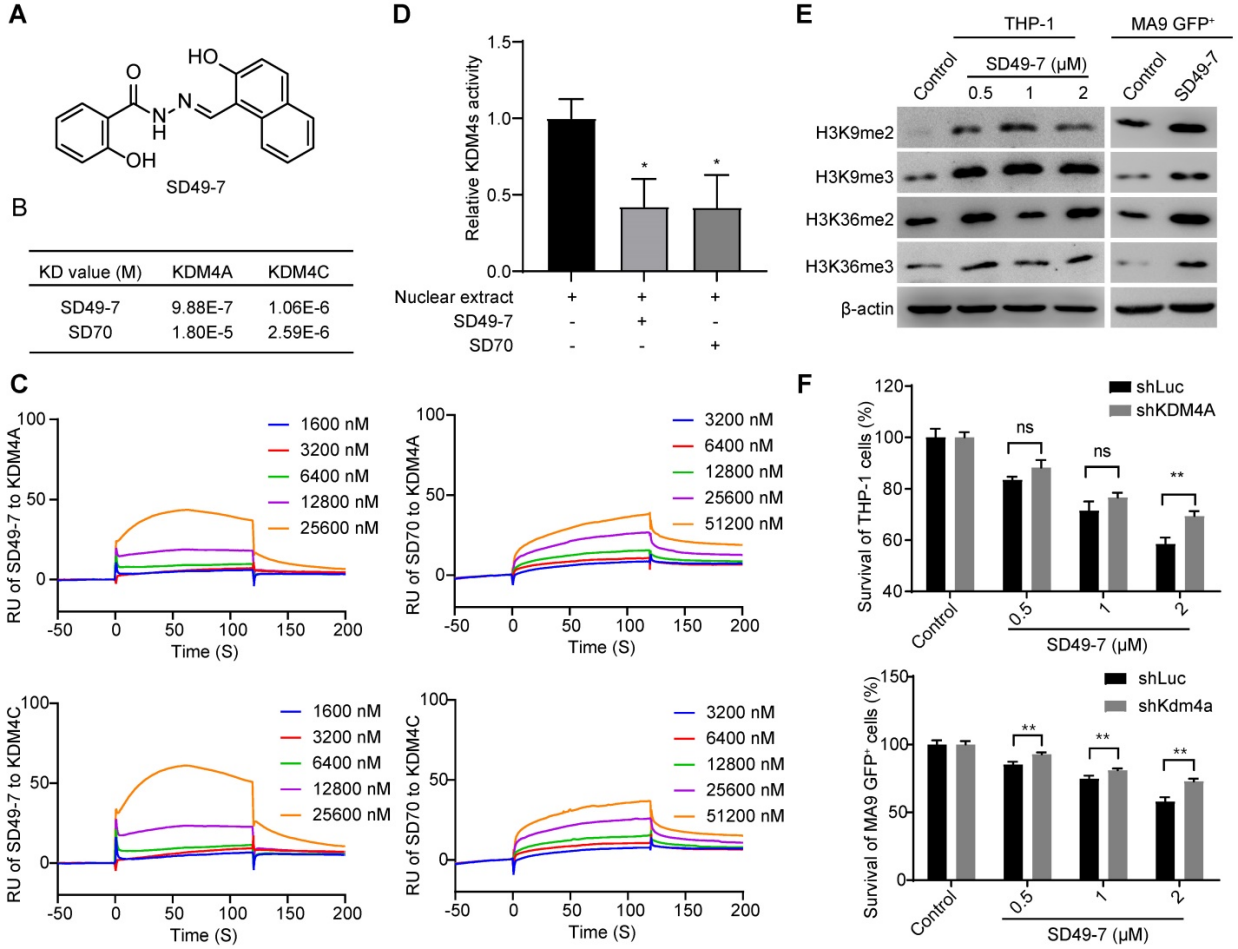
** Specificity of a small molecule inhibitor SD49-7 of KDM4s. (A)** Structure of SD49-7. **(B)** KD value of SD49-7 or SD70 interaction with KDM4A or KDM4C, respectively, as detected by SPR assay. **(C)** SPR results of the binding activity of KDM4A or KDM4C at various concentrations of SD49-7 or SD70. **(D)** Western blot analysis of the levels of tri- or di- methylation of H3K9 or H3K36 regulated by SD49-7 in THP-1 (left panel) and MLL-AF9 GFP^+^ cells (right panel, SD49-7 concentration: 1 µM). β-actin was used as a loading control, and the experiment was repeated three times. **(E)** Effect of SD79-7 (1 µM) and SD70 (1.75 µM) on demethylase kinetics was evaluated by JMJD2 demethylase activity *in vitro*. THP-1 cell nuclear extract without inhibitors was used as a control. **(F)** Apoptosis of THP-1 cells (left panel) and MLL-AF9 leukemia cells (right panel) triggered by SD49-7 treatment for 24 h when KDM4A was silenced by shRNAs (n = 3). Data were normalized to the control shLuc or shKDM4A group. ns. no significance, *p < 0.05, **p < 0.01, by unpaired Student's* t*-test, error bars denote mean ± SD.

8-hydroxyquinolines have been reported as cell-active histone demethylase inhibitors 25. It has been reported that the 8-hydroxyquinoline derivative SD70 could attenuate prostate cancer cells by targeting KDM4C 11, 12. We acquired a series of known and/or potential KDM4 inhibitors 25, which were synthesized and purified using an established method 26 and evaluated their activities against adriamycin-resistant cell lines HL-60/ADR and K562/ADR for the first-round screen (Table S1). Next, the cytotoxicity of 9 candidates from the first-round screen was tested in adriamycin-resistant cell lines and three additional leukemia cell lines, including K562, HL-60, and THP-1 (Table 1). SD49-7 was the most outstanding compound that could inhibit sensitive and drug-resistant leukemia cells (Figure 2A). Compared to the reported compound SD70, SD49-7 exhibited stronger leukemia cell inhibition. The IC_50_ value of SD49-7 against three human leukemia cell lines was much lower than SD70 (Figure S2A). Since the KDM4 family was shown to trigger di- or tri-demethylation of H3K9 and H3K36 22, we tested the effect of SD49-7 on KDM4 substrates. We observed that SD49-7 could dose-dependently induce H3K9me2/3 and H3K36me2/3 accumulation in THP-1 cells and also increased H3K9me2/3 and H3K36me2/3 levels in mouse MLL-AF9 cells (Figure 2D). These data indicated that SD49-7 might inhibit KDM4 and lead to the accumulation of H3K9 and H3K36. Since KDM4B showed no significant difference in AML cells, we tested the effect of SD49-7 on the expression of KDM4A and KDM4C. We found that the mRNA level of KDM4A was slightly decreased by SD49-7 in both THP-1 and MLL-AF9 leukemia cells (Figure S2B), whereas it decreased KDM4A and KDM4C protein levels only in THP-1 cells, but not in MLL-AF9 leukemia cells (Figure S2C-D). Since SD49-7 increased the methylation levels of H3K9 and H3K36 in mouse leukemia cells, these data led us to investigate the interaction of SD49-7 with KDM4 proteins.

We performed surface plasma resonance (SPR) to validate the interactions between SD49-7 or SD70 and KDM4A or KDM4C *in vitro.* KDM4A and KDM4C proteins were immobilized on a CM5 chip individually and were exposed to various dilutions of SD49-7 or SD70. SD49-7 bound to KDM4A or KDM4C with a dissociation constant of KD = 9.88×10^-7^ mol/L or KD = 1.06×10^-6^ mol/L, respectively, and SD70 bound to KDM4A or KDM4C with a dissociation constant of KD = 1.80×10^-5^mol/L or 2.59×10^-6^mol/L, respectively (Figure 2B). These data showed an 18.2-fold higher affinity of SD49-7 to KDM4A than SD70. Both SD49-7 and SD70 bound to KDM4A or KDM4C in a dose-dependent manner (Figure 2C). The SPR results also suggested that SD70 preferentially bound to KDM4C rather than KDM4A while SD49-7 could directly bind to KDM4A and KDM4C. Moreover, KDM4 demethylase activity assays showed that both SD49-7 and SD70 decreased the demethylase activity of KDM4s in THP-1 nuclear extract (Figure 2D). We further noticed that tri- and di- methylation levels of H3K9 and H3K36 were increased upon treatment with SD49-7 in THP-1 and MLL-AF9 GFP^+^ cells (Figure 2E, Figure S2E). To further verify KDM4s as the target of SD49-7, we assessed the sensitivity of KDM4 inhibitor in KDM4A knocked-down THP-1 and MLL-AF9 GFP^+^ cells against SD49-7. The result showed that the apoptosis induced by SD49-7 decreased by silence of KDM4A in THP-1 cells or MLL-AF9 GFP^+^ cells, suggesting the therapeutic effects of SD49-7 be dramatically compromised in KDM4A knockdown leukemic cells (Figure 2F). Taken together, compared to SD70, SD49-7 could efficiently inhibit KDM4A and KDM4C, resulting in leukemia suppression.

### Pharmacological inhibition of KDM4s suppresses leukemia development

**Figure 3:**
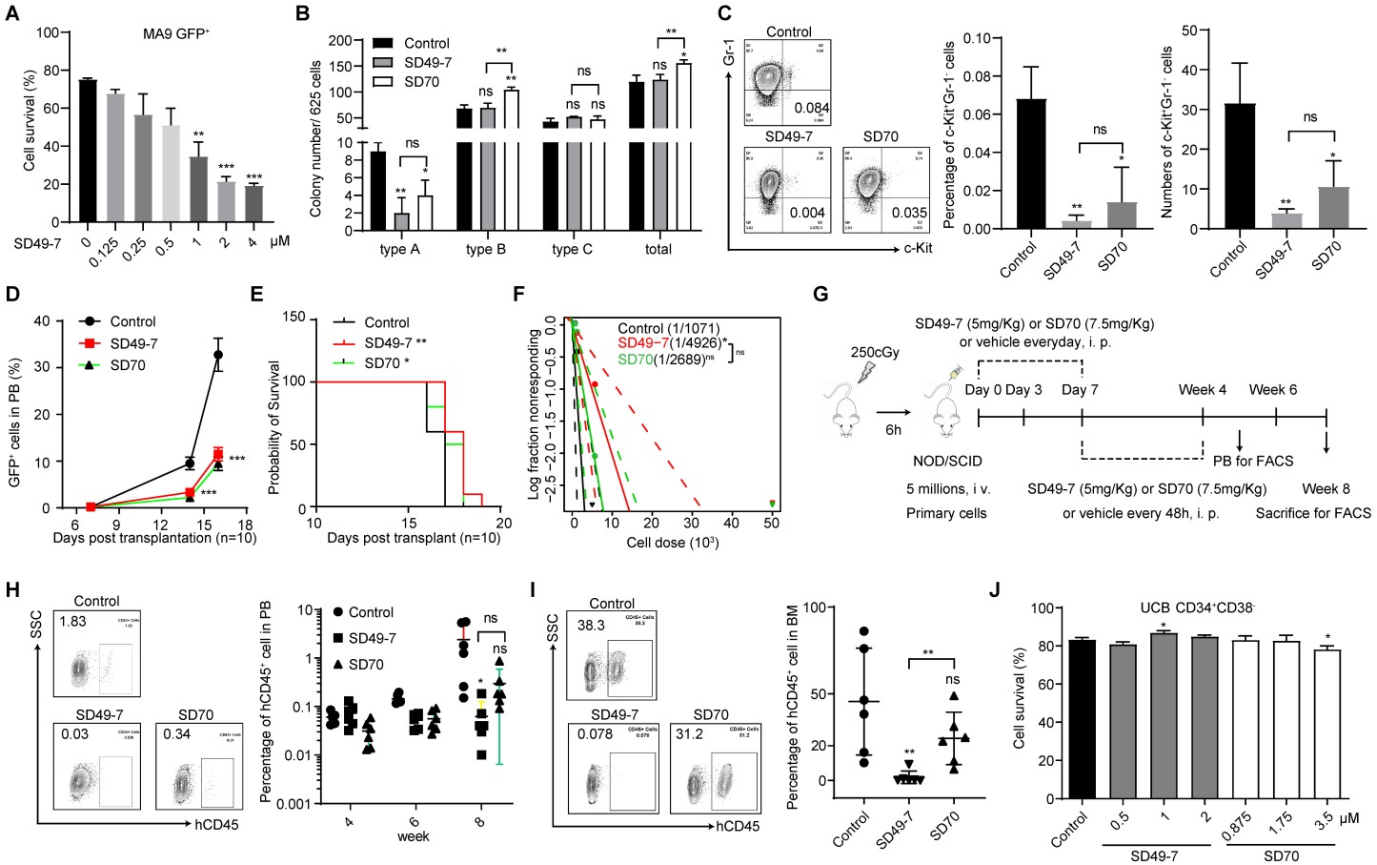
** Pharmacological inhibition of KDM4s suppresses leukemia development. (A)** Apoptosis of MLL-AF9 leukemia cells triggered by SD49-7 treatment for 24 h (n = 3). Data were normalized to the control. **(B)** Colony numbers of MLL-AF9 leukemia cells following 24 h pre-treatment with SD49-7 (1 µM) or SD70 (1.75 µM). 625 cells were seeded per well in a 24-wellplate after the treatment (n = 3). **(C)** Percentage (middle panel) and absolute (right panel) numbers of c-Kit^+^Gr-1^-^ cell population in MLL-AF9 leukemia cells after treatment for 24 h with SD49-7 (1 µM) or SD70 (1.75 µM), n = 3. Representative flow cytometric results are shown in the left panel. **(D)** MLL-AF9 leukemia cell proliferation measured by cell counting after SD49-7 (1 µM) or SD70 (1.75 µM) treatment for 24 h (n = 10). **(E)** Survival plot representing the percentage of surviving mice injected with MLL-AF9 leukemia cells treated with 1 µM SD49-7 or 1.75 µM SD70 (n = 10). **(F)** Limiting dilution transplantation of MLL-AF9 leukemia cells following 24-h treatment with SD49-7 (1 µM) or SD70 (1.75 µM). Cell dose: 50,000, 5,000, 500, 50, n = 8. ns. no significance *p < 0.05 by chi-square test. **(G)** Workflow of the PDX model-related experimental design. **(H)** Percentage of hCD45^+^ cells (right panel) in PB of the PDX model after week 4, 6, and 8 detected by flow cytometry (n = 6). Representative flow cytometric results at week 8 are shown in the left panel. **(I)** Percentage of hCD45^+^ cell (right panel) in BM were detected by flow cytometry in the PDX model (right panel, n = 6). Representative flow cytometric results are shown in the left panel. **(J)** Apoptosis of CD34^+^CD38^-^ UCB cells treated with SD49-7 (0, 0.5, 1, or 2 µM) or SD70 (0, 0.8755, 1.75, or 3.5 µM) for 24 h (n = 3). Vehicle was used as a negative control. ns. no significance, *p < 0.05, **p < 0.01, by unpaired Student's* t*-test, error bars denote mean ± SD.

We investigated the therapeutic potential of targeting KDM4s in AML and examined the impact of SD49-7 in a mouse MLL-AF9 leukemia model 21. SD49-7 dose-dependently induced apoptosis of MLL-AF9 GFP^+^ cells (Figure 3A). The colony formation ability of MLL-AF9 GFP^+^ cells was also decreased by treatment with SD49-7 (Figure 3B). Next, we tested the effects of SD49-7 in mouse LSCs, in which the percentage and the absolute number of the c-Kit^+^Gr-1^-^ population were markedly inhibited by SD49-7 (Figure 3C). Although there was no significant difference between the SD49-7 and SD70 groups, the average LSC percentage levels and absolute numbers were lower in the SD49-7 group (Figure 3C). We also used the THP-1 cell line to evaluate the leukemia inhibition activity of SD49-7 and found that SD49-7 could significantly suppress cell growth (Figure S3A), induce cell apoptosis (Figure S3B), differentiation (Figure S3C), and cell-cycle arrest (Figure S3D). These data demonstrated the anti-leukemia activity of SD49-7 *in vitro*.

Next, we performed an *ex vivo* transplantation assay. Compared to the control mice, the percentage of GFP^+^ cells in peripheral blood was significantly decreased by SD49-7 and SD70 treatment (Figure 3D), and the life span of recipient mice was slightly extended by SD49-7 and SD70 (Figure 3E). The anti-leukemogenic potential of SD49-7 was then detected by a limiting dilution assay (LDA). The SD49-7 group showed an approximately 5-fold decrease of functional LSCs (1 in 4925) compared with the control group (1 in 1071), whereas the SD70 group (1 in 2088) exhibited about a 2-fold decrease of functional LSCs (Figure 3F). These data suggested that SD49-7 attenuated the leukemogenic ability of leukemia cells. To further validate the pharmacological cytotoxicity of SD49-7, a patient-derived tumor xenograft (PDX) model was established, as illustrated in Figure 3G. The FACS analysis data of peripheral blood showed a significant decrease of hCD45^+^ cells in SD49-7 mice compared with the control group (Figure 3H). Moreover, SD49-7 treatment markedly decreased the percentage of hCD45^+^ cells in bone marrow at week 8, whereas SD70 treatment did not significantly inhibit the growth of human leukemia xenografts (Figure S3E, Figure 3I); half of the 6 control group recipients achieved over 50% engraftment, but none of the SD49-7 or SD70 group recipients achieved over 50% human leukemia cell xenograft engraftment. The engraftment rates of SD49-7-treated mice were lower than 10%, while 5 of 6 SD70 treated mice achieved over 10%, in which 4 of 6 mice reached 20-50% (Figure S3F). These data indicated that both SD49-7 and SD70 suppressed leukemia development *in vivo,* and SD49-7 showed a stronger effect inhibiting leukemia progression than SD70.

We used human cord blood MNCs to explore the effect of SD49-7 and SD70 on normal human hematopoietic cells. The results showed that treatment with either SD49-7 or SD70 did not promote the apoptosis of human CD34^+^CD38^-^ cells *in vitro* (Figure 3J). Colony formation assays showed that SD49-7 treatment decreased the numbers of BFU-E, whereas SD70 decreased GM, BFU-E, and the total colony numbers (Figure S3G). We observed that the morphology of main organs, such as the intestine, lung, liver, kidney, and heart, was not affected by SD49-7 or SD70 (Figure S3H). These results indicated that SD49-7 exerted a weaker effect on normal human hematopoietic cells than SD70. Given that SD49-7 exhibited an 18.2-fold higher affinity for KDM4s relative to SD70 (Figure 2B), our data supported that KDM4 is an outstanding target in attenuating leukemia cells without affecting normal hematopoiesis. Indeed, KDM4 inhibitor SD49-7 strongly suppressed leukemia progression with only minimal effects on normal human hematopoietic cells.

### KDM4s trigger MDM2/p21^CIP1^ in leukemia cells

It has been reported that the KDM4/JMJD2 histone demethylases are required for hematopoietic stem cell maintenance and the expression of two downstream genes, Taf1b and Nom1, are critical for KDM4-mediated hematopoietic cell maintenance 27. Here, we tested the expression of Taf1b and Nom1 in normal and leukemia cells following treatment with SD49-7. We found that KDM4 inhibitors decreased mRNA levels of both Taf1b and Nom1 in mouse c-Kit^+^ cells and human CD34^+^ cells (Figure S4A). However, these inhibitors did not decrease the expression of Taf1b and Nom1 in both MLL-AF9 GFP^+^ cells and THP-1 cells (Figure S4B), indicating that KDM4 regulated different downstream genes in leukemia and normal cells.

**Figure 4:**
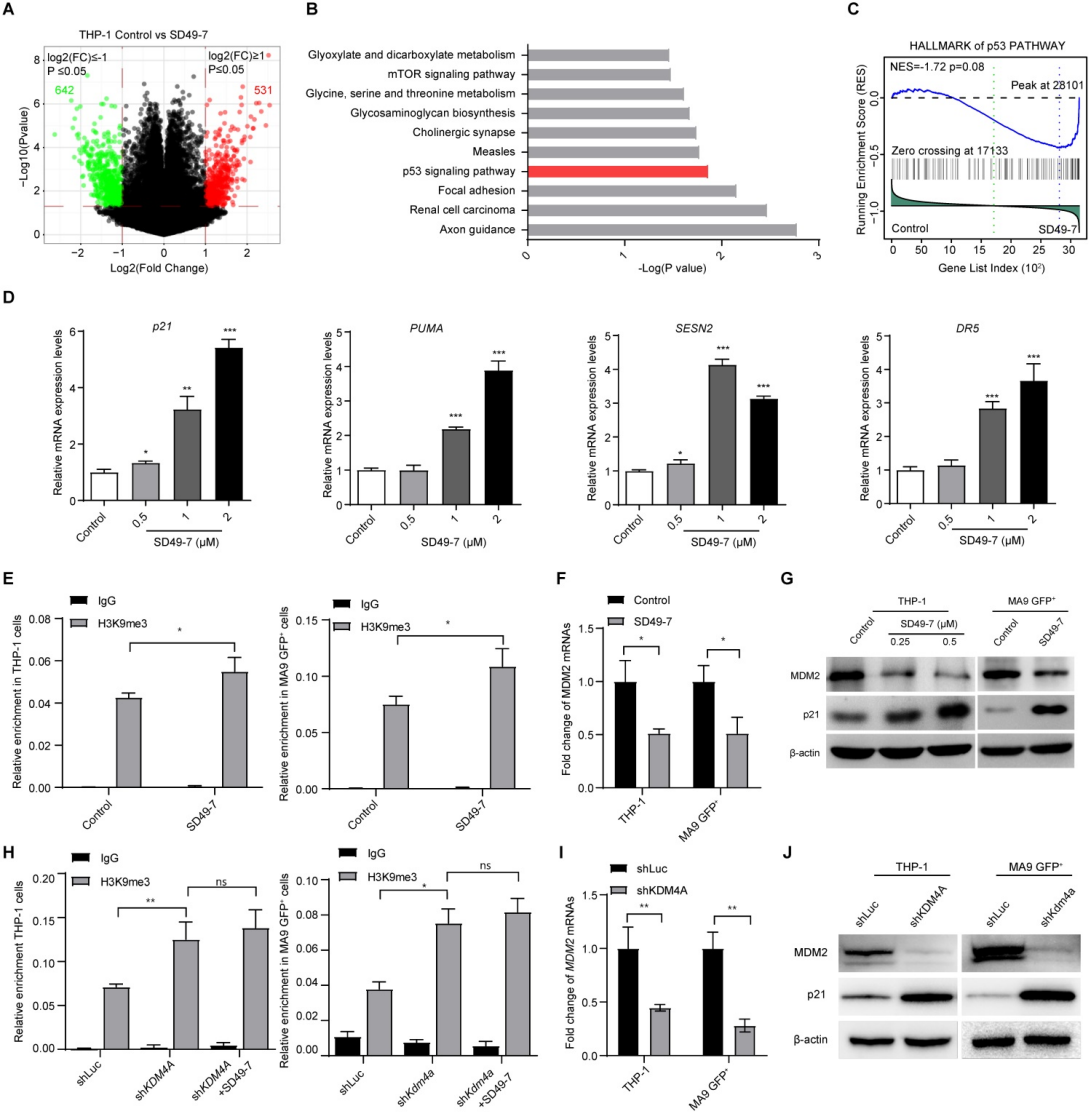
** KDM4A triggers MDM2 and p21 in leukemia cells. (A)** Volcano plot showing the genes up-regulated or down-regulated by SD49-7 in THP-1 cells by cDNA microarray. Cut off: fold-change > 2, p ≤ 0.05. **(B)** Enrichment of KEGG analysis of differentially expressed genes. **(C)** GSEA enrichment of the hallmark p53 pathway. **(D)** Fold-change of mRNA levels of p53 and its targets (p21, PUMA, SESN2, and DR5) mediated by 24 h SD49-7 treatment in THP-1 cells (n = 3). Data were normalized to the control. **(E)** Enrichment of the MDM2 promoter in THP-1 cells (left panel) and MLL-AF9 leukemia cells (right panel) after a 24 h treatment of 1 µM SD49-7 (n = 3). IgG was used as a negative control. **(F)** Fold-change of MDM2 mRNA levels in THP-1 cells and MLL-AF9 leukemia cells following 24-h treatment with 1 µM SD49-7 (n = 3). Data were normalized to the control. **(G)** Western blot analysis of MDM2, p53, and p21 protein levels in THP-1 cells and MLL-AF9 leukemia cells following 24-h treatment with SD49-7 (1 µM in MLL-AF9 GFP^+^ cells). β-actin was used as a loading control. Experiments were repeated three times. **(H)** Enrichment of the MDM2 promoter in THP-1 cells (left panel) and MLL-AF9 leukemia cells (right panel) when KDM4A was abolished with shRNAs (n = 3). IgG was used as a negative control. **(I)** Fold-change of MDM2 mRNA levels in THP-1 cells and MLL-AF9 leukemia cells when KDM4A was abolished by shRNAs (n = 3). Data were normalized to the control. **(J)** Western blot analysis of MDM2 and p21 protein levels in THP-1 cells and MLL-AF9 leukemia cells when KDM4A was abolished by shRNAs. β-actin was used as a loading control. Experiments were repeated three times. Vehicle was used as a negative control. ns. no significance, *p < 0.05, **p < 0.01, ***p < 0.001, by unpaired Student's* t*-test, error bars denote mean ± SD.

To assess the mechanism by which KDM4 deficiency suppressed leukemia progression, we used SD49-7 to inhibit KDM4s and analyzed wholegenome gene expression in THP-1 cells treated with SD49-7. Total RNA was extracted from cells treated with SD49-7 or vehicle, and the expressed transcripts were analyzed by a wholegenome gene expression chip. Our data showed that 531 and 642 genes were significantly up-regulated and down-regulated, respectively, by SD49-7 treatment (Figure 4A, Figure S4C). KEGG analysis of differentially expressed genes showed regulation of several signaling pathways by SD49-7 in which p53-related genes (MDM2, p21^CIP1^, PUMA) were dramatically influenced (Figure 4B). We also analyzed the wholegenome gene expression in the control and SD49-7 groups by GSEA with the geneset “Hallmark p53 pathway-related genes” and the results showed that p53-related genes were enriched in the SD49-7 group (Figure 4C).

Next, we validated the effect of SD49-7 on the expression of p53-related genes. We observed up-regulation of mRNAs of p53-related genes, such as p21^CIP1^, PUMA, SESN2, and DR5, by SD49-7 treatment (Figure 4D). Since the accumulation of methylated H3K9 was reported to be involved in inhibiting nearby genes 28, we assessed H3K9 methylation levels on the *MDM2* promoter, which was reported to act as an E3 ligase regulator 29. ChIP-PCR assays showed a statistically significant increase in H3K9me3 levels by SD49-7 treatment in both THP-1 and MLL-AF9 GFP^+^ cells (Figure 4E), indicating that SD49-7 may regulate MDM2 expression by modulating H3K9 methylation on the *MDM2* promoter. Indeed, MDM2 mRNA levels were decreased by SD49-7 treatment in both THP-1 cells and MLL-AF9 GFP^+^ cells (Figure 4F). MDM2 regulates the p21^CIP1^ protein level in cancers 30-32. Western blotting showed that MDM2 was decreased while p21^CIP1^ was increased by SD49-7 treatment in both THP-1 and MLL-AF9 GFP^+^ cells (Figure 4G, Figure S4D). These data suggested that SD49-7-induced accumulation of methylated H3K9 decreased the MDM2 level and activated p21^CIP1^.

To rule out the off-target effect of SD49-7, we assessed the regulatory effect of KDM4A on p53-related genes. Consistent with SD49-7 treatment, knockdown of KDM4A by shRNA increased the accumulation of H3K9me3 on the MDM2 promoter in both THP-1 and MLL-AF9 GFP^+^ cells (Figure 4H), and MDM2 mRNA levels in both THP-1 cells and MLL-AF9 GFP^+^ cells were decreased by KDM4A knockdown (Figure 4I). Moreover, we found that SD49-7 could not increase the enrichment of H3K9me3 at the MDM2 promoter in KDM4A knockdown THP-1 and MLL-AF9 GFP^+^ cells (Figure 4H). MDM2 protein levels were down-regulated by the KDM4A knockdown, and the p53-related genes, such as p21^CIP1^, were up-regulated by KDM4A silencing in both THP-1 and MLL-AF9 GFP^+^ cells (Figure 4J, Figure S4E), suggesting that KDM4A regulated MDM2-p53 signaling in leukemia cells. Thus, our data suggested that KDM4A deficiency by SD49-7 or shRNA enhanced trimethylation of H3K9 at the MDM2 promoter and then activated p21^CIP1^ due to the decreased expression of MDM2.

## Discussion

Numerous reports have indicated that LSCs are a source of drug resistance and relapse in leukemia treatment 33. Since LCSs are characterized by self-renewal and resistance to cytotoxic chemotherapeutics, the underlying regulatory mechanisms and discovery of drugs targeting LSCs have attracted much attention. In this context, epigenetics is believed to be significant in regulating the development and progression of leukemia 34. Elucidating the epigenetic regulation mechanisms is significant for targeting LSCs by pharmaceutical compounds and preventing relapse in leukemia treatment.

The mutations of several epigenetic regulators, such as TET2, IDH1, IDH2, DNMT3a, and EZH2, have been well-recognized in leukemogenesis 35. However, the regulatory role of epigenetics in LSCs needs to be further elucidated. The KDM4 family, including KDM4A, KDM4B, KDM4C, and KDM4D, demethylating H3K9me2/3 and H3K36me2/3, is considered an important regulator in AML 11, 14. Cheung et al. found that KDM4C plays a key role in the development of multiple MLL and non-MLL leukemias 11. Another study by Agger et al. found that knocking out *Kdm4c* alone had no apparent effect on leukemogenesis, and significant inhibition of AML was observed when *Kdm4a, b, and c* were knocked out simultaneously 14. However, the study did not investigate the effect of deletion of KDM4A alone.

In the present study, we found that depletion of KDM4A by either shRNA or its inhibitor SD49-7 could sufficiently impede leukemogenesis, indicating that KDM4A may be a more suitable target in impairing leukemia cells. We also observed that SD49-7 showed stronger leukemia cell inhibition than SD70, which preferentially binds to KDM4C. Thus, the high affinity of SD49-7 to KDM4A and KDM4C led to this difference. Our data were consistent with the findings of Jin et al. that SD70 acted as an inhibitor of KDM4C, but its effect on KDM4A was not apparent 12. Thus, we provided evidence that among the KDM4 family, KDM4A and KDM4C are the most efficient potential targets in leukemia treatment. During drug design, the cytotoxicity to leukemia cells and the side effects on normal hematopoietic cells are critically important. Epigenetic regulators are not considered appropriate targets in leukemia treatment because of their global effects on neoplastic and normal cells and epigenetic drug design requires careful fine-tuning of these targets. Thus far, several epigenetic drugs with good efficacy have entered clinical trials, including the IDH inhibitor AG-221 36, BET inhibitor OTX015 37, HDAC inhibitor pracinostat 38, LSD1 inhibitor GSK2879552 39, and DOT1L inhibitor pinometostat 40. We have shown that the small molecule compound SD49-7, which targets KDM4A and KDM4C, could inhibit leukemia progression. Most importantly, SD49-7 exhibited minimal cytotoxicity to human CD34^+^CD38^-^ UCB cells. Consistent with these findings, knockdown of KDM4A did not affect the survival of human CD34^+^ UCB cells. These data indicated that KDM4A and KDM4C might serve as specific targets, and SD49-7 may be an efficacious and safe candidate for leukemia treatment. Thus, the KDM4A and KDM4C structures merit further attention in future KDM4 drug design. And more accurately measurement, such as using KDM4A knockout cells for assessing of the safety of targeting KDM4A and SD49-7 effect on the long-term self-renewal of HSCs and the normal hematopoiesis *in vivo* could be further determined.

KDM4/JMJD2 histone demethylases were reported to be essential for hematopoietic stem cell maintenance 27. In this study, we found that the expression of Taf1b and Nom1, downstream of KDM4s, was not affected by SD49-7 in MLL-AF9 GFP^+^ and THP-1 cells, indicating that KDM4 regulated different downstream genes in leukemia and normal cells. The KDM4 family is considered an important regulator in AML 11, 14. However, the mechanism of KDM4 in leukemia regulation remains unclear. We demonstrated that KDM4A and KDM4C, but not KDM4B, regulated leukemia progression. Depleting KDM4A and KDM4C by either shRNA or SD49-7 enhanced trimethylation of H3K9 occupying the promoter of MDM2 and activated p21^CIP1^ by decreasing MDM2 expression, inhibiting the stemness and proliferation, and activating apoptosis of leukemia cells. Previously, therapeutic use of the MDM2-p53 inhibitor APG-115 obtained FDA orphan drug designation for the treatment of gastric cancer 41, SD49-7, which triggers MDM2 inhibition *via* KDM4s, represents a promising candidate for developing new targeted drugs in leukemia treatment. Our work also provides new insights into the mechanism of KDM4 in leukemia regulation and novel ideas for targeting LSCs.

In summary, our study emphasized the regulatory role of KDM4s in LSCs by providing evidence that the depletion of KDM4s by the small molecule inhibitor SD49-7 could sufficiently inhibit leukemogenesis without impairing normal hematopoietic cells. Mechanistically, we showed that the KDM4 inhibitor triggered the function of a p53-related gene in LSC regulation. Thus, a novel KDM4 inhibitor could be a promising candidate for leukemia therapy by selectively eradicating LSCs. Our work provided innovative insights into the mechanism of epigenetic regulation of KDM4 in LSCs for epigenetic drug design to target LSCs.

## Materials and Methods

### Patient samples

Primary samples of AML patients (n = 15) were provided by the Institute of Hematology and Blood Diseases Hospital (Tianjin, China). Umbilical cord blood samples (n = 15) of healthy donors were acquired by Shandong Qilu stem cell engineering Co., Ltd. This study was carried out in accordance with the Declaration of Helsinki and approved by the Ethics Review Board of the Institute of Hematology and Blood Diseases Hospital and the Chinese Academy of Medical Sciences (Tianjin, China).

### Mice

Mice were bred in a specific-pathogen-free animal core facility with free access to food and water. All animal protocols were approved by the Animal Care and Use Committee of the State Key Laboratory of Experimental Hematology.

### Lentivirus infection

Primary MLL-AF9 GFP^+^ leukemia cells, provided by the Tao Cheng laboratory 21, were cultured in 15% fetal bovine serum (FBS), 10 ng/mL mIL-6, 10 ng/mL mIL-3, 50 ng/mL mSCF, and 1% penicillin-streptomycin and plated into RetroNectin (Takara)-pre-coated 24-well plates. Polybrene (5 µg/mL) and lentivirus (final concentration of 100 multiplicity of infection (MOI), Shanghai Genechem Co, LTD.) were added to each well. After the plates were centrifuged at 1,800 rpm at 33 °C for 90 min, the cells were cultured at 37 °C in a humidified incubator. After 48 h, GFP^+^mCherry^+^ cells were sorted with a flow cytometer and were used for the following experiments.

CD34^+^ hUCB cells were isolated by immunomagnetic methods using the MACS CD34 progenitor cell isolation kit (Miltenyi Biotec). Isolated human UCB CD34^+^ cells were resuspended in IMDM (Gibco) supplemented with 10% FBS (Gibco), 100 ng/mL human SCF (PeproTech), 100 ng/mL human TPO (PeproTech), and 100 ng/mL human Flt3L (PeproTech) at a concentration of 1 × 10^5^ cells/mL. Next, cells were plated into RetroNectin precoated 24-well plates. Polybrene (at a final concentration of 5 µg/mL) and lentivirus (at a final concentration of 100 MOI) were added to each well. After the plates were centrifuged at 1,800 rpm at 33 °C for 90 min, the cells were cultured at 37 °C in a humidified incubator. After 48 h, the GFP^+^ cells were sorted with a flow cytometer and were used for the following experiments.

### qRT-PCR

Total RNAs of MLL-AF9 or hUCB cells were extracted using TRIzol reagent (Invitrogen) according to manufacturer's instructions. After RNA quality control by Nanodrop 2000 (Nanodrop Technologies), 5 μg of total RNA was reverse transcribed into cDNA using One-Step gDNA Removal and cDNA Synthesis SuperMix (TransGen Biotech) according to the manufacturer's instruction. Anchored oligo(dT)20 primer was used to obtain cDNA. Quantitative RT‐PCR was performed in 10 μL quantitative PCR reactions in triplicate using TB Green Premix Ex Taq (Takara) following the manufacturer's protocol. The PCR was run in an ABI QuantStudio 6 (Thermo Fisher Scientific) real-time fluorescence quantitative PCR instrument with a cycling condition of 95 °C for 2 min, followed by 40 cycles of 95 °C for 5 sec and 60 °C for 34s. The expression of target transcripts was standardized to GAPDH, and the relative expression of target transcripts was calculated according to the 2^-ΔΔCt^ method. Primers used in this study are shown in Table S2.

### Western blotting

Total cellular proteins from murine primary MLL-AF9 leukemia cells or hUCB cells were lysed with RIPA buffer (Thermo Fisher Scientific), resolved on 12% SurePAGE Gels (GenScript) and transferred to PVDF membranes (Merck Millipore). The antibodies used are listed in Table S3. Western Lightning Plus ECL (Perkin Elmer) reagents were used for fluorescence production, and ChemiDoc chemiluminescence imager (Bio-Rad) was used for fluorescence detection to visualize the proteins. The gray value of blots standardized to β-actin was quantified by Image J software.

### Apoptosis, proliferation, differentiation, and cell cycle analysis

The indicated number of cells were seeded in 24-well plates, cultured, and treated with various compounds for 24h. An apoptosis analysis kit (Tianjin Sungene Biotech Co., Ltd.) was applied to detect the apoptosis status of cells by flow cytometry (LSR II, BD Biosciences). Cell differentiation was measured by the fluorescence intensity of CD11b. Cells were resuspended in PI/RNase staining buffer for further cell cycle analysis. For proliferation analysis, cells were cocultured (with SD49-7 or an equivalent volume of DMSO) for 72h and were counted every 24h by the trypan blue exclusion cell counting method.

### Colony-forming unit assay

THP-1 cells or umbilical cord blood-derived CD34^+^ cells were plated in methylcellulose medium (MethoCult H4434, StemCell Technologies) according to the manufacturer's instructions. Murine primary MLL-AF9 derived leukemia cells were cultured in MethoCult M3434 (StemCell Technologies) methylcellulose medium. Colonies were counted and recorded under a microscope (Operetta CLS™, PerkinElmer or NIKON) after 7-12 days of incubation. For the serial colony-forming unit assay, colonies were collected and readjusted for cell density as before, and the colony-forming unit assay procedure was repeated. The number and morphological features of colonies were recorded 7 days later.

### Cell culture and cytotoxicity assay

HL-60, K562, HL-60/ADR, K562/ADR, and THP-1 cell lines were grown in suspension in RPMI 1640 medium (Gibco) supplemented with 2mM L-glutamine, 10% heat-inactivated FBS (Gibco), 1% penicillin/streptomycin (Thermo Fisher Scientific) in a humidified 5% CO_2_ incubator at 37 °C. A stock solution of SD49-7 and SD70 at a concentration of 20mM was prepared by dissolving the compound in sterile DMSO and stored at -20 °C until use.

MTT assays were used to detect the cytotoxicity of compounds. Leukemia cells were seeded in 96-well plates at a density of 5×10^3^ cells/well and treated with different compounds at a concentration gradient for 72 h. After treatment, MTT was added at a final concentration of 0.5 mg/mL and allowed to react with cells for 4 h. The formazan precipitate was dissolved in DMSO, and absorbance was read at 570 nm using a microplate reader (Synergy H4, BioTek). The 50% inhibitory concentration (IC_50_) value was determined by GraphPad Prism 8 project.

### Surface plasma resonance (SPR) array

The SPR system (Biacore T200, GE Healthcare Sciences, Pittsburgh, PA, USA) was used to measure the binding of SD49-7 or SD70 to KDM4A and KDM4C, respectively. Briefly, KDM4A or KDM4C proteins were dissolved in 10 mM sodium acetate buffer (pH 4.0) to a final concentration of 100 µg/mL and then were immobilized on CM5 chips (GE Healthcare, Pittsburgh, PA, USA) using a standard amine coupling procedure. The running buffer was PBS (pH 7.4) with 0.005% P20 surfactant. Various dilutions of compounds were used and the binding of SD49-7 or SD70 to KDM4A or KDM4C was analyzed with Biacore T200 Evaluation Software 3.1 to get the KD value.

### KDM4 demethylase activity assay

Nuclear extraction kit (Epigentek) was applied to isolate nuclear proteins from THP-1 cells according to the manufacturer's instructions. The total yield was up to 100 μg per 10^7^ cells. Nuclear extracts were used as the enzyme source to measure demethylase activity.

Demethylase kinetics of SD79-7 and SD70 was evaluated by the JMJD2 demethylase activity assay kit (Epigentek). Enzymatic reactions were set up for blank and samples with nuclear extracts in the presence or absence of inhibitors (1 μM SD79-7 and 1.75 μM SD70) and incubated with the substrate and assay buffer at 37 °C for 120 min. Subsequently, the wells were washed and incubated with the capture antibody at room temperature for 60 min followed by incubation with a detection antibody at room temperature for 10 min. The amount of demethylated products was colorimetrically detected by a microplate reader (Bio-Tek) at 450 nm with an optional reference wavelength of 655 nm. The KDM4s demethylase activity was calculated using the following formula: Inhibition % = (1-(Inhibitor Sample OD - Blank OD)/(No Inhibitor Sample OD - Blank OD)) × 100%.

### *Ex vivo* and limiting dilution assays

Murine primary MLL-AF9 leukemia cells expressing GFP were treated with compounds at the indicated concentration for 24 h, and then collected and resuspended in cold PBS. For the *ex vivo* assay, viable cells (2×10^5^ cells/0.2 mL PBS) were injected into the tail vein of syngeneic 18-19g female C57BL/6J mice. The mice were sacrificed 21 days later to detect the leukemia burden in major hematopoietic tissues. For the limiting dilution assay, 50,000, 5,000, 500 or 50 cells were injected into the tail vein of C57BL/6J mice. The number of recipients that developed leukemia and died was recorded to calculate the LSC frequencies according to Poisson statistics with ELDA software 42.

### Xenograft transplantation experiments

Primary mononuclear cells (MNCs) isolated from a relapsed AML patient were injected into sub-lethally irradiated (250 cGy) NOD/SCID mice *via* tail vein 3 days before injection with SD49-7 (5 mg/kg), SD70 (7.5 mg/kg), or vehicle (PEG300: dextrose 5% in water (D5W) = 3:1) intraperitoneally (i.p.) for 5 consecutive days and then every 2 days for 3 weeks. About 8 weeks after transplantation, mice were sacrificed to analyze the engraftment efficiency in bone marrow by flow cytometry (LSR II, BD Biosciences).

### Whole-genome gene expression chip analysis

THP-1 cells were treated with SD49-7 for 24 hours. The cells were lysed to isolate RNA, and gene expression was detected using a whole-genome gene expression chip (Genergy). Data analysis was performed to identify critical pathways. Differentially expressed genes were cut off with fold-change > 2 or < 0.5, p ≤ 0.05. KEGG analysis (DAVID; https://david.ncifcrf.gov/) 43 was used to detect changes in molecular functions, biological processes, and cellular components induced by CSSD9. Gene set enrichment analysis (GSEA) was performed using GSEA_4.0.3 software (http://www.gsea-msigdb.org/gsea/index.jsp). The geneset of HALLMARK P53 PATHWAY (http://www.gsea-msigdb.org/gsea/msigdb/cards/HALLMA RK _P53_ PATHWAY.html) was used to assess the effect of SD49-7 on p53 signaling.

### Chromatin immunoprecipitation assays

Chromatin immunoprecipitation (ChIP) assays were performed using the Enzymatic Chromatin IP Kit (Cell Signaling Technology) according to the manufacturer's instructions. THP-1 cells were lysed after culturing for 24 hours with or without SD49-7 treatment. Protein/DNA complexes were immunoprecipitated by H3K9me3 antibodies, using normal rabbit IgG as a negative control. The primers used for PCR amplification are shown in Table S2.

### Statistical analysis

Analyses were carried out by GraphPad Prism v8.0 with Student's-*t* test, and the Kaplan-Meier method and Log-rank test were used for survival analysis. Quantitative results are presented as the mean ± standard deviation (SD), and a p-value < 0.05 was considered to be statistically significant.

Other details of **Reagents** or **Resources** can be found in **Supplementary Materials.**

## Highlights

- KDM4A depletion inhibits leukemogenesis and avoids the impairment of normal hematopoietic cells.
- A specific KDM4 inhibitor, SD49-7, selectively eradicates LSCs without impairing normal hematopoietic cells.
- KDM4 inhibition deregulates MDM2 and elevates p21^CIP1^.

## Supplementary Material

Supplementary figures and tables.

## Figures and Tables

**Table 1:** Screen of KDM4 inhibitors in leukemia cell lines

Compound	IC_50_ (μM)				
	THP-1	HL-60	HL-60/ADR	K562	K562/ADR
A70	1.20 ± 0.09	0.84 ± 0.09	2.052	1.42 ± 0.16	1.216
SD70	1.76 ± 0.11	5.17 ± 0.03	5.545	2.14 ± 0.34	2.942
SD70-11	3.69 ± 0.30	1.18 ± 0.20	3.518	2.81 ± 0.24	2.636
SD70-22	0.98 ± 0.18	0.43 ± 0.07	2.246	0.97 ± 0.12	2.062
SD70-25	0.75 ± 0.07	0.23 ± 0.03	1.983	0.62 ± 0.09	1.428
SD49-7	0.19 ± 0.04	0.12 ± 0.02	2.079	0.60 ± 0.13	1.365
SD49-8	0.31 ± 0.08	0.26 ± 0.03	3.947	0.82 ± 0.11	1.53
GHDM1526	> 10	1.101 ± 0.03	> 10	3.25 ± 0.32	6.311
GHDM1530	2.31 ± 0.26	1.34 ± 0.29	> 10	2.77 ± 0.17	4.362
