# Supplementary material for: KDM4 inhibitor SD49-7 attenuates leukemia stem cell via KDM4A/MDM2/p21CIP1 axis
Source: Theranostics. 2022 Jun 21;12(11):4922–34. doi: 10.7150/thno.71460 (PMC9274755; doi:10.7150/thno.71460)
Supplement: Supplementary file 1 — Supplementary figures and tables. [file thnov12p4922s1.pdf]

Supplementary materials

Supplementary figure legends

Figure S1. (A) Relative mRNA levels of KDM4A in MLL-AF9 leukemia cells transfected with three different KDM4A shRNAs (n = 3). (B) Relative flow cytometry image of GFP^+ $mCherry^+$ cells after sorting (left panel) and the effect validation in protein levels (right panels). The most efficient shRNA was used. Western blot analysis was repeated three times. (C) Cell cycle analysis of MLL-AF9 leukemia cells transfected with KDM4A shRNA (n = 3). (D) Relative mRNA levels of KDM4A in THP-1 cells transfected with three different KDM4A shRNAs (n = 3). (E) Relative flow cytometry image of GFP^+ cells after sorting (left panel) and the effect validation in protein levels (right panels). The most efficient shRNA was used. Western blot analysis was repeated three times. (F) Apoptosis of THP-1 cells in shLuc and shKDM4A groups (n = 3). (G) Cell cycle analysis of THP-1 cells transfected with KDM4A shRNA (n = 3). (H) Relative fluorescence intensity of CD11b in THP-1 cells (n = 3). Representative flow cytometry image is shown in the right panel. (I) Colony numbers (left) and representative microscopy images of colony formation (right) of shLuc and shKDM4A groups (n = 3). 1,000 THP-1 cells per well were seeded into a 24 well plate. Scale bar = 50 μm. (J) Efficiency of lentiviral virus targeting KDM4A or KDM4C measured by flow cytometry (left panel) and mRNA levels (right) by RT-PCR (n = 3). (K) Colony numbers of $CD34^+$ UCB cells with knockdown of KDM4A or KDM4C (n = 3). GEMM, colony-forming unit-granulocyte, erythroid, macrophage, megakaryocyte; CFU-GM, colony-forming unit-granulocyte, macrophage; BFU-E, burst-forming unit-erythroid; CFU-E, colony-forming unit-erythroid. (L) Typical images of representative morphologies of various types of colonies as indicated. *$p < 0.05$, **$p < 0.01$, ***$p < 0.001$, by unpaired Student's *t*-test, error bars denote mean ± SD.

Figure S2. (A) IC_{50} of SD49-7 and SD70 in HL-60, K562 and THP-1 cell lines, respectively. (B) Relative mRNA levels of KDM4A in THP-1 cells (left panel) and MLL-AF9 leukemia cells (right panel) treated with SD49-7 (n = 3). (C) Western blot analysis of KDM4A in THP-1 cells (left panel) and MLL-AF9 leukemia cells (right panel) treated with SD49-7. β-actin served as a loading control. The experiment was repeated three times. (D) Relative protein expression

levels relative to Figure S2C. (E) Relative protein expression levels relative to Figure 2E. ns. no significance, *$p < 0.05$, **$p < 0.01$, ***$p < 0.001$, ****$p < 0.0001$, by unpaired Student's *t*-test, error bars denote mean ± SD.

Figure S3. (A) Proliferation curve of THP-1 cells treated with SD49-7, by cell counting (n = 3). (B) Apoptosis of THP-1 cells treated with SD49-7 (n = 3). (C) Relative fluorescence intensity of CD11b in THP-1 cells (n = 3). Data are normalized to control. (D) Cell cycle analysis of THP-1 cells treated with SD49-7 (n = 3). (E) Flow cytometry image of PDX model BM. (F) Percentage of different transplantation rate. (G) Left panel: Colony numbers of $CD34^+$ UCB cells, treated with 1 μM SD49-7 or 1.75 μM SD70 (n = 3). GEMM, colony-forming unit-granulocyte, erythroid, macrophage, megakaryocyte; CFU-GM, colony-forming unit-granulocyte, macrophage; BFU-E, burst-forming unit-erythroid; CFU-E, colony-forming unit-erythroid. Right panel: Typical images of representative morphologies of various types of colonies as indicated. (H) Typical images of organs of NOD/SCID mice in the PDX model. *$p < 0.05$, **$p < 0.01$, ***$p < 0.001$, by unpaired Student's *t*-test, error bars denote mean ± SD.

Figure S4. (A) Relative mRNA levels of TAF1B and NOM1 in human and mouse HSPCs, treated with 1 μM SD49-7 or 1.75 μM SD70 (n = 3). (B) Relative mRNA levels of TAF1B and NOM1 in human and mouse leukemia cells, treated with 1 μM SD49-7 or 1.75 μM SD70 (n = 3). (C) Heatmap of genes in GSEA analysis. (D) Relative protein expression levels of Figure 4G. (E) Relative protein expression levels relative to Figure 4J. ns. no significance, *$p < 0.05$, **$p < 0.01$, ***$p < 0.001$, ****$p < 0.0001$ by unpaired Student's *t*-test, error bars denote mean ± SD.

Supplementary Tables

Table S1. First round screen of KDM4 inhibitors

Compound	IC_{50} (μM)		Compound	IC_{50} (μM)	
	HL-60/ADR	K562/ADR		HL-60/ADR	K562/ADR
A70	2.052	1.216	GHDM1202	> 10	> 10
SD70	5.545	2.942	GHDM1203	> 10	> 10
SD70-1	> 10	> 10	GHDM1204	> 10	> 10
SD70-2	> 10	> 10	GHDM1205	> 10	> 10
SD70-3	> 10	7.941	GHDM1206	> 10	> 10
SD70-4	> 10	7.548	GHDM1207	> 10	> 10
SD70-5	> 10	> 10	GHDM1208	> 10	> 10
SD70-6	> 10	> 10	GHDM1209	> 10	> 10
SD70-7	> 10	> 10	GHDM1210	> 10	> 10
SD70-8	> 10	> 10	GHDM1211	> 10	> 10
SD70-9	> 10	> 10	GHDM1212	> 10	> 10
SD70-10	> 10	> 10	GHDM1213	> 10	> 10
SD70-11	3.518	2.636	GHDM1214	> 10	> 10
SD70-14	> 10	9.848	GHDM1215	> 10	> 10
SD70-15	> 10	> 10	GHDM1216	> 10	> 10
SD70-16	> 10	9.849	GHDM1217	> 10	> 10
SD70-17	5.154	4.407	GHDM1218	> 10	> 10
SD70-18	6.142	3.812	GHDM1219	> 10	> 10
SD70-19	> 10	> 10	GHDM1220	> 10	> 10
SD70-20	> 10	3.832	GHDM1401	> 10	> 10
SD70-21	> 10	3.117	GHDM1402	> 10	> 10
SD70-22	2.246	2.062	GHDM1403	> 10	> 10
SD70-23	> 10	> 10	GHDM1404	> 10	> 10
SD70-24	7.904	4.168	GHDM1501	> 10	> 10
SD70-25	1.983	1.428	GHDM1502	> 10	> 10
SD49	9.076	6.593	GHDM1503	> 10	> 10
SD49-1	5.746	7.111	GHDM1504	> 10	> 10
SD49-2	6.63	4.308	GHDM1505	> 10	> 10
SD49-3	> 10	> 10	GHDM1506	> 10	> 10

SD49-4	> 10	> 10	GHDM1507	> 10	> 10
SD49-5	> 10	> 10	GHDM1508	> 10	> 10
SD49-6	5.249	5.286	GHDM1509	> 10	> 10
SD49-7	2.079	1.365	GHDM1510	> 10	> 10
SD49-8	3.947	1.53	GHDM1511	> 10	> 10
SD49-9	> 10	> 10	GHDM1512	> 10	> 10
SD49-10	> 10	> 10	GHDM1513	> 10	> 10
SD49-11	> 10	> 10	GHDM1514	> 10	> 10
SD49-12	> 10	> 10	GHDM1515	> 10	> 10
GHDM1000	> 10	> 10	GHDM1516	> 10	> 10
GHDM1001	> 10	> 10	GHDM1517	> 10	> 10
GHDM1002	> 10	> 10	GHDM1518	> 10	> 10
GHDM1003	> 10	> 10	GHDM1519	> 10	> 10
GHDM1004	> 10	> 10	GHDM1520	> 10	> 10
GHDM1005	> 10	> 10	GHDM1521	> 10	> 10
GHDM1006	> 10	> 10	GHDM1522	> 10	> 10
GHDM1007	> 10	> 10	GHDM1523	> 10	> 10
GHDM1008	> 10	> 10	GHDM1524	> 10	> 10
GHDM1009	> 10	> 10	GHDM1525	> 10	> 10
GHDM1010	> 10	> 10	GHDM1526	> 10	6.311
GHDM1011	> 10	> 10	GHDM1527	> 10	> 10
GHDM1101	> 10	> 10	GHDM1528	> 10	> 10
GHDM1102	> 10	> 10	GHDM1529	> 10	> 10
GHDM1103	> 10	> 10	GHDM1530	> 10	4.362
GHDM1104	> 10	> 10	GHDM1531	> 10	> 10
GHDM1105	> 10	> 10	GHDM1601	> 10	> 10
GHDM1201	> 10	> 10	GHDM1602	> 10	> 10

Table S2. Primers used in real-time PCR

**Primers**	**Forward (5'-3')**	**Reverse (5'-3')**
KDM4A-mRNA	CGTGGGTCCACGTTTCATG	GCCCGTTCTTATGCTTGCTAAT
KDM4B-mRNA	TTCAATCACGGGTTCAACTGCG	GGTCTTCGGGCTTCGGCTTC
KDM4C-mRNA	CTGTGCAAAGTGCTGCGTAC	TTCTGGGACATTAGTGAATCGA
p53-mRNA	GGCCCACTTCACCGTACTAA	GTGGTTTCAAGGCCAGATGT

p21-mRNA	GATTAGCAGCGGAACAAGGAGT	TACAGTCTAGGTGGAGAAACGGG
PUMA-mRNA	TACGAGCGGCGGAGACAAGAG	GCGGGTGCAGGCACCTAATT
SESN2-mRNA	GGCCTGCACCCTGACTACTTTACC	CGCCAGCAACTTGTTGGATCTCG
DR5-mRNA	CCCAAGACCCTTGTGCTCG	GGGACTTAGCTCCACTTCACCT
MDM2-mRNA	GCAGTGAATCTACAGGGACG	GTGCATTTCCAATAGTCAGCTAAG
GAPDH-mRNA	CATCACCATCTTCCAGGAGCG	TGACCTTGCCCACAGCCTTG
hMDM2-Promoter	TTCATCCAGGGTCAAGCACTG	ACTCAGTTGATTTAAGTTGATTGCC
mMdm2-Promoter	CCATCCTGCTCATGCCCC	GCCTTGGGAGATGAAGTAGCC
hTAF1B-mRNA	CTCGAGGAGGCGGAAGAGTT	TGGGCCTACTCCAAGGTTCT
mTaf1b-mRNA	GTGAAAGCGTTCAGAGACCG	TGTCTCAGCCCCCTGTTGAT
hNOM1-mRNA	ACTGCAGAGAGCTTTGGTCC	CAGGTGCGTATGGTGACTGT
mNOM1-mRNA	TCCTACGGAGGGTGTTGACT	TTGGGTTGTCAGACACTCTTGA

Table S3. Antibody used in present study

Antibody	SOURCE	IDENTIFIER
Anti-mouse c-Kit-APC	eBioscience	Cat# 17-1171-82
Anti-human Gr1-PE-cy7	eBioscience	Cat# 25-5931-82
Anti-human CD45-FITC	BD	Cat# 555482
Anti-mouse CD11b-PE	BD	Cat# 555388
β-actin	Sigma-Aldrich	Cat# A5316
H3K9me2	CST	Cat# 4658s
H3K9me3	CST	Cat# 13969
H3K36me2	CST	Cat# 2901
H3K36me3	CST	Cat# 4909
KDM4A	Abcam	Cat# ab191433
MDM2	Immunoway	Cat# YT2692
p21	Immunoway	Cat# YM3453

Figure S1

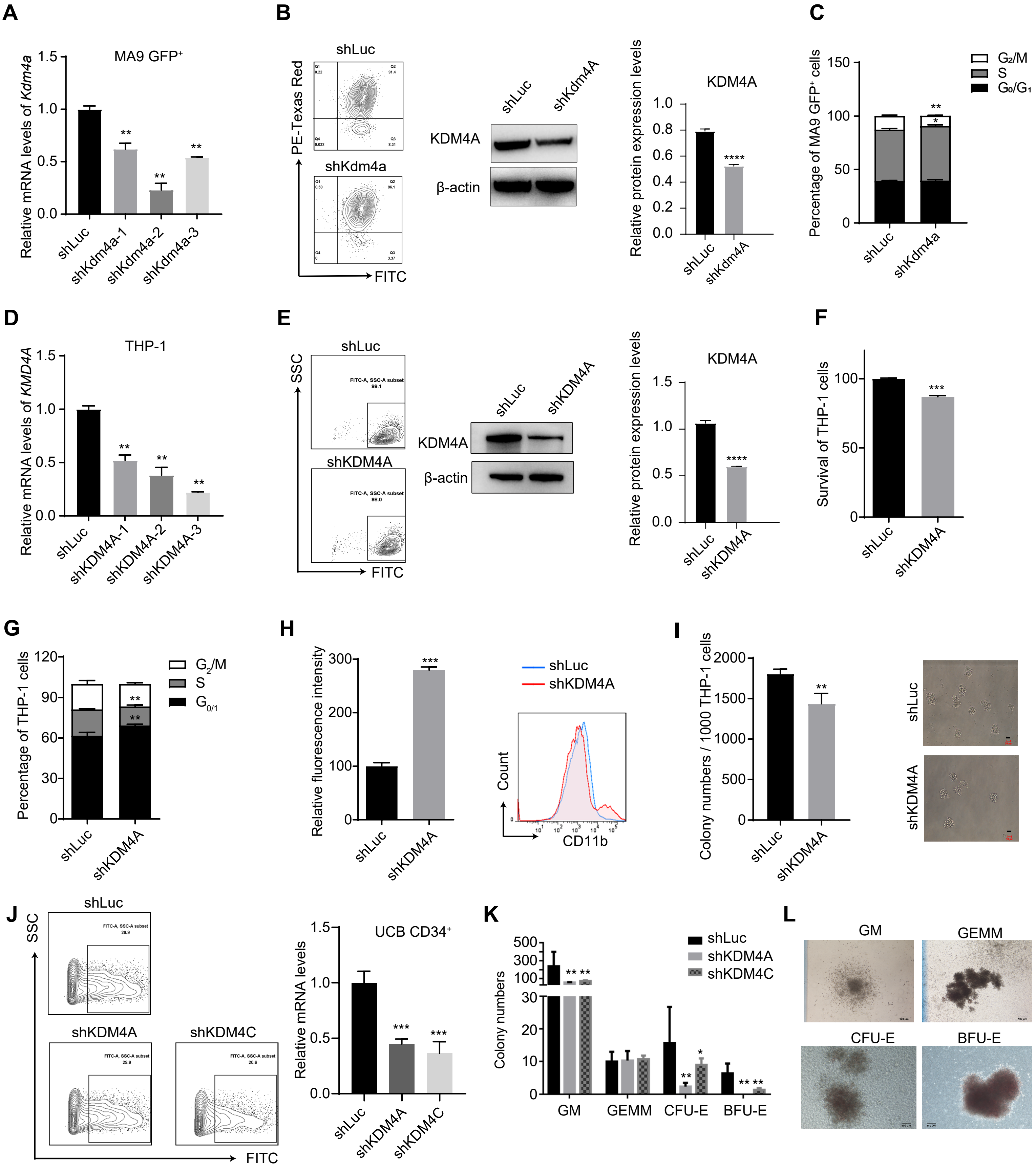
A
MA9 GFP+
Relative mRNA levels of Kdm4a
shLuc
shKdm4a-1
shKdm4a-2
shKdm4a-3
B
shLuc
shKdm4a
PE-Texas Red
FITC
KDM4A
β-actin
shKdm4a
Relative protein expression levels
C
G2/M
S
G0/G1
Percentage of MA9 GFP+ cells
D
THP-1
Relative mRNA levels of KMD4A
shKDM4A-1
shKDM4A-2
shKDM4A-3
E
SSC
shKDM4A
F
Survival of THP-1 cells
G
Percentage of THP-1 cells
H
Relative fluorescence intensity
Count
CD11b
I
Colony numbers / 1000 THP-1 cells
J
UCB CD34+
Relative mRNA levels
shKDM4C
K
Colony numbers
GM
GEMM
CFU-E
BFU-E
L

Figure S2

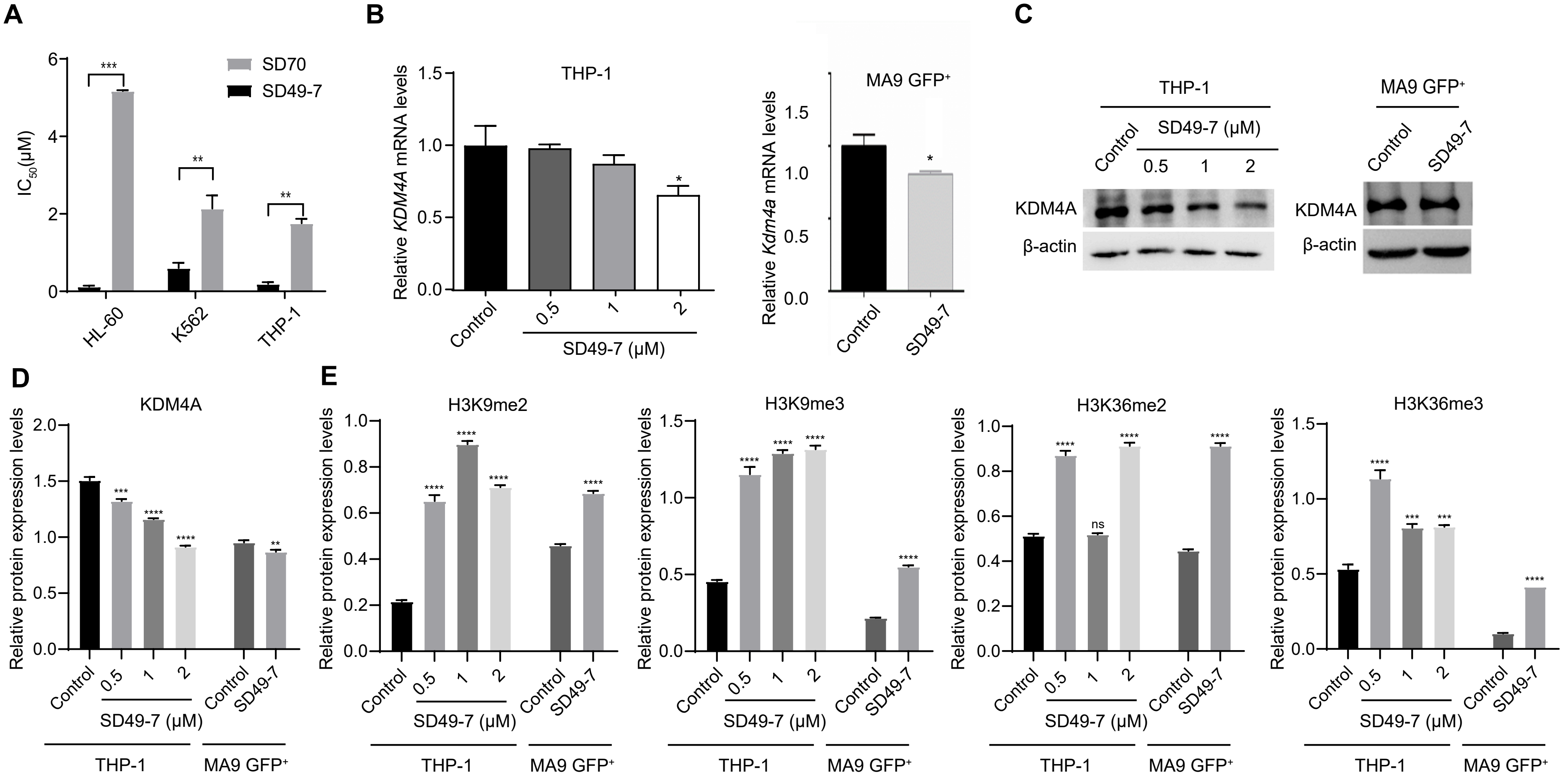
A
B
C
D
E
SD70
SD49-7
IC50(μM)
HL-60
K562
THP-1
Relative KDM4A mRNA levels
Relative Kdm4a mRNA levels
MA9 GFP+
Control
SD49-7 (μM)
KDM4A
β-actin
Relative protein expression levels
H3K9me2
H3K9me3
H3K36me2
H3K36me3

Figure S3

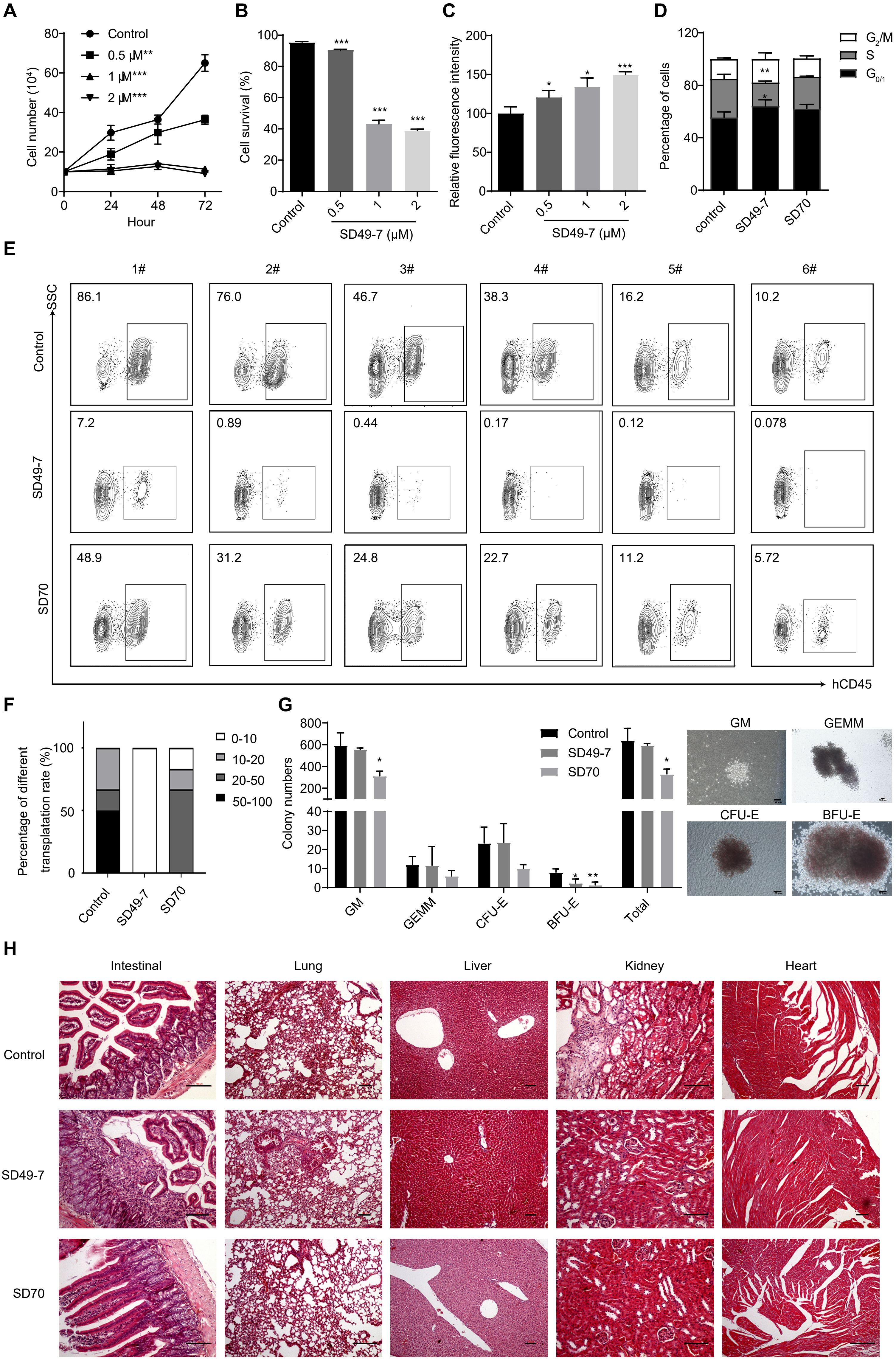

A
Cell number (10^4)
Hour
Control
0.5 μM**
1 μM***
2 μM***
B
Cell survival (%)
Control
SD49-7 (μM)
C
Relative fluorescence intensity
D
Percentage of cells
G2/M
S
G0/1
control
SD49-7
SD70
E
1#
2#
3#
4#
5#
6#
SSC
hCD45
86.1
76.0
46.7
38.3
16.2
10.2
7.2
0.89
0.44
0.17
0.12
0.078
48.9
31.2
24.8
22.7
11.2
5.72
F
Percentage of different transplatation rate (%)
0-10
10-20
20-50
50-100
G
Colony numbers
GM
GEMM
CFU-E
BFU-E
Total
H
Intestinal
Lung
Liver
Kidney
Heart

Figure S4

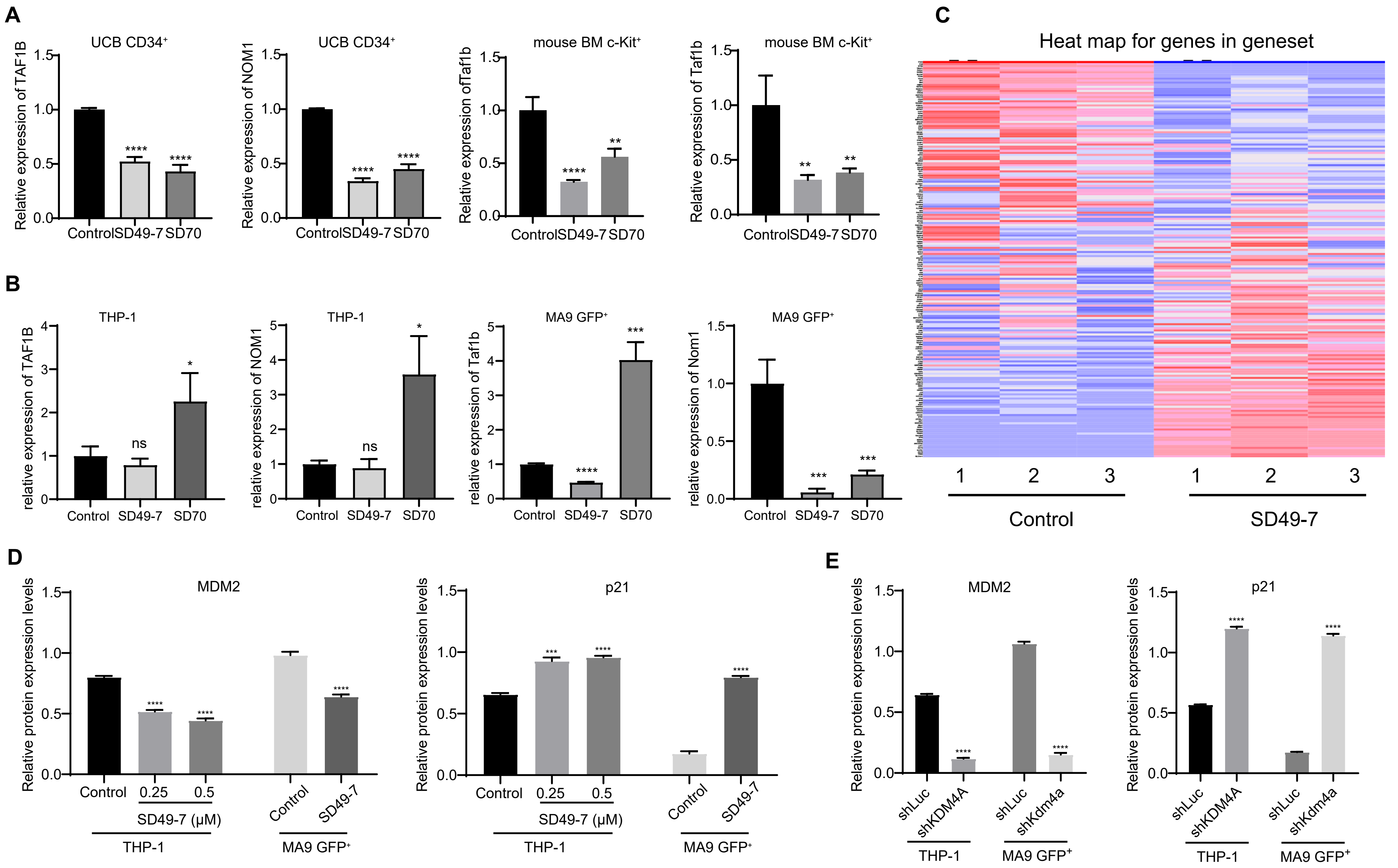

A
UCB CD34⁺
Relative expression of TAF1B
Relative expression of NOM1
mouse BM c-Kit⁺
Relative expression ofTaf1b
Relative expression of Taf1b
Control
SD49-7
SD70
B
THP-1
relative expression of TAF1B
relative expression of NOM1
MA9 GFP⁺
relative expression of Taf1b
relative expression of Nom1
ns
C
Heat map for genes in geneset
1
2
3
Control
SD49-7
D
MDM2
p21
Relative protein expression levels
0.25
0.5
SD49-7 (μM)
THP-1
MA9 GFP⁺
E
shLuc
shKDM4A
shKdm4a
